# Effect of blood contamination on marginal adaptation of cold ceramic and MTA angelus: a scanning electron microscopic study

**DOI:** 10.1186/s12903-023-03437-6

**Published:** 2023-09-30

**Authors:** Fatemeh Mokhtari, Jalil Modaresi, Abbas Bagheri

**Affiliations:** 1 Department of Endodontics, Dental School of Shahid Sadoughi University of Medical Sciences, Yazd, Iran; 2 School of Dentistry, Imam Reza Street, Yazd, 8914815667 Iran

**Keywords:** Dental marginal adaptation, Endodontics, Electron scanning Microscopy, MTA-Angelus, Root Canal filling materials

## Abstract

**Background:**

This study aimed to assess the effect of blood contamination on marginal adaptation of cold ceramic (CC) and mineral trioxide aggregate (MTA) Angelus using scanning electron microscopy (SEM).

**Methods:**

This in vitro experimental study was conducted on 24 extracted single-rooted human teeth. After cleaning and shaping, the root canals were filled with lateral compaction technique. The apical 3 mm of the roots was cut, and cavities with 3 mm depth were created at the apex. The teeth were randomly assigned to two group (n = 12) for the application of CC and MTA Angelus as retrograde filling materials. CC and MTA Angelus were prepared by mixing the powder with blood, and applied in the cavities. After 24 h, their marginal adaptation to the canal walls was assessed by SEM. Data were statistically analyzed by t-test (alpha = 0.05).

**Results:**

The mean marginal gap was 8.98 μm in the CC, and 16.26 μm in the MTA Angelus group; this difference was statistically significant (P < 0.001).

**Conclusions:**

The present in vitro study revealed that following complete blood contamination of powder, CC showed significantly superior marginal adaptation than MTA Angelus as shown by SEM assessment.

## Introduction

Microleakage of stimulants into the periapical tissue is a major cause of endodontic treatment failure [1–3]. An ideal root filling material should be able to optimally seal the communication paths between the root canal system and periapical tissues. Also, it should be biocompatible, biologically safe (no toxicity, no carcinogenicity), insoluble in tissue fluids, and radiopaque for easier radiographic detection. Moreover, it should have optimal dimensional stability and easy application, and its seal should not be compromised by moisture exposure. The abovementioned characteristics are independent of the purpose of application as an orthograde or retrograde root filling material. Nonetheless, none of the currently available root filling materials possess all of the aforementioned properties [4, 5].

Several materials have been proposed for root end filling [6, 7]; of which, mineral trioxide aggregate (MTA) is commonly used as the gold-standard for the purpose of comparison since it often brings about promising results, and has favorable properties such as biocompatibility and no toxicity [8–11].

Cold ceramic (CC) is another material recently introduced for root end filling, which has shown favorable properties [12, 13]. The primary chemical constituents comprising CC predominantly encompass calcium oxide, silicon oxide, barium oxide, and sulfur trioxide, collectively comprising approximately 93% of its overall composition [14]. A previous study compared the tissue reaction to CC and MTA and showed that both of them were well tolerated by the tissues [12]. Calcium hydroxide is the main constituent of CC, which is biocompatible [12]. An in vitro study indicated that CC had a stronger sealing ability than amalgam [13]. To prepare CC, its powder should be mixed with its respective liquid. It primarily sets within 15 min; however, its final setting occurs within 24 h. To date, CC has shown favorable properties [14].

Several methods are used for assessment of microleakage and marginal adaptation of root filling materials and their sealing potential, such as the bacterial leakage models, dye penetration techniques, electrochemical methods, radioisotopes, fluid filtration technique, and scanning electron microscopy (SEM) [15, 16]. and Achieving an adequate bond strength at the interface between materials and dentin holds utmost significance in guaranteeing an efficient seal of the root canal system, ultimately aiming to avert or reduce microleakage [17]. SEM is among the most accurate techniques for assessment of marginal adaptation by measurement of marginal gap after root canal treatment and evaluation of the sealing capacity of root filling materials [12, 18, 19].

Several previous studies assessed the microleakage, marginal adaptation, and properties of MTA and CC in different environments [8, 13, 20, 21]. However, the effect of blood contamination on their sealing properties by mixing the powder with blood, instead of the respective liquid, has not been previously investigated. The ideal condition for MTA and CC is that the powder of both materials should be mixed with the liquid suggested by the manufacturer without any contamination from blood [4, 8, 14, 22]. However, in this study, we investigate the opposite end of this spectrum, which involves the complete mixing of the powder with blood. Thus, this study aimed to assess the effect of blood contamination (by mixing the powder with blood, instead of the respective liquid) on marginal adaptation of CC and MTA Angelus using SEM. The null hypothesis of the study was that the marginal adaptation of CC and MTA Angelus would be the same after blood contamination.

## Methods

This in vitro, experimental study was conducted on 24 single-rooted human teeth extracted for purposes not related to this study such as periodontal disease, and irreparable coronal caries. The study protocol was approved by the ethics committee of Yazd Shahid Sadoughi University of Medical Sciences (IR.SSU.DENTISTRY.REC.1401.027).

### Sample size

The sample size was calculated to be 12 in each group assuming a standard deviation of 2.87 for the MTA and 2.01 for the CC group for marginal adaptation, and assuming 3-score difference in marginal adaptation of the two groups with 95% confidence interval and 80% study power using PASS 15 software.

### Eligibility criteria

Single-canal extracted human teeth with no history of previous endodontic treatment, and no crack, resorption, fracture, or caries in the root were included. All roots had a mature apex, straight canal, and no calcification.

### Methodology

**Fig. 1:**
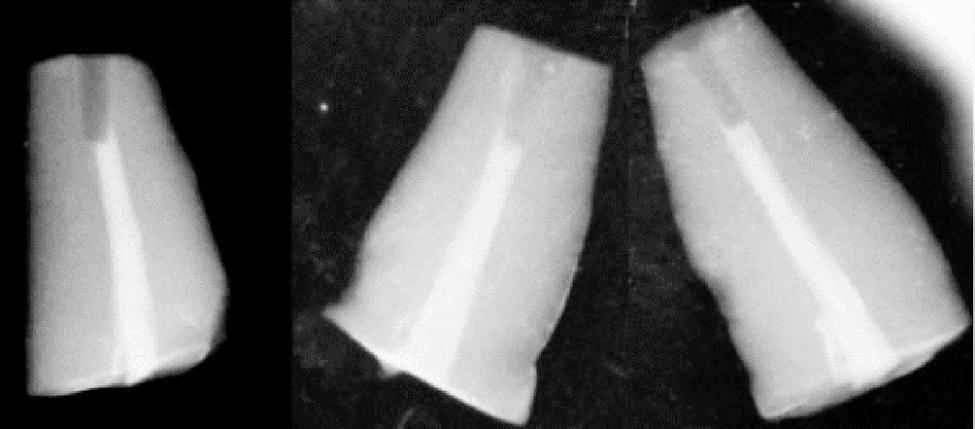
Radiographic view of the roots after cutting the apical 3 mm and creating a cavity with 3 mm depth (by gutta-percha removal) at the root end using an ultrasonic scaler

The teeth were immersed in 5.25% sodium hypochlorite (Nikdarman, Iran) for 30 min to eliminate the superficial debris. The crowns were cut at the cementoenamel junction in order to obtain root samples of uniform length, measuring 15 mm, by utilizing a low-speed handpiece equipped with a safe-sided diamond disk and employing water coolant. A #15 K-file (Mani Inc., Tochigi-Ken, Japan) was inserted into the root canal until it reached the apical foramen. The working length was determined as such [22]. The root canals were cleaned and shaped using a set of ProTaper instruments(Dentsply Maillefer, Ballaigues, Switzerland), consisting of Shaping Files 1 and 2 (S1 and S2) and the Finishing Files 1 through 3 (F1^F3). The root canals were irrigated with 1ml of sodium hypochlorite 5.25% (Nikdarman, Iran) during the cleaning and shaping process after each instrument and a 20 ml final flush with 5.25% sodium hypochlorite [23] and then For smear layer removal, the canals were rinsed saline, followed by 10 ml 17% EDTA for 3 min (Nikdarman, Iran), and a subsequent rinse with saline. Final rinse was performed with 10ml 5.25% sodium hypochlorite [23]. The root canals were dried with paper point (Meta, Meta Dental Co., Seoul, South Korea) and obturated with gutta-percha (Diadent, Seoul, Korea) and AH26 sealer (Dentsply, DeTrey, Konstanz, Germany) by the cold lateral compaction technique a #30,0.02 GP cone used as the master cone and the rest of canal obturated with #20,0.02 and #15,0.02 along side of #20,0.02 and #15,0.02 finger spriders (Dentsply Maillefer, Ballaigues, Switzerland). The teeth were incubated at 37 °C temperature and 100% humidity for 5 days. Next, the apical 3 mm of the roots was cut with 90-degree angle relative to the longitudinal axis of the root with a diamond disc (D and Z, Darmstadt, Germany). A cavity with 3 mm depth was created at the apex by gutta-percha removal using an ultrasonic scaler (Woodpecker UDS-K LED ultrasonic scaler, Guilin Woodpecker Medical Instrument, China) and conformation radiographies were taken immediately after that (Fig. 1). The teeth were then randomly assigned to two groups (n = 12) as follows:

Group 1: The cavity at the root end was filled with MTA after mixing the MTA Angelus powder (Angelus, Londrina, PR, Brazil) with human blood.

Group 2: The cavity at the root end was filled with CC after mixing the CC powder (sarv javid modares,YAZD,IRAN) with human blood.

**Fig. 2:**
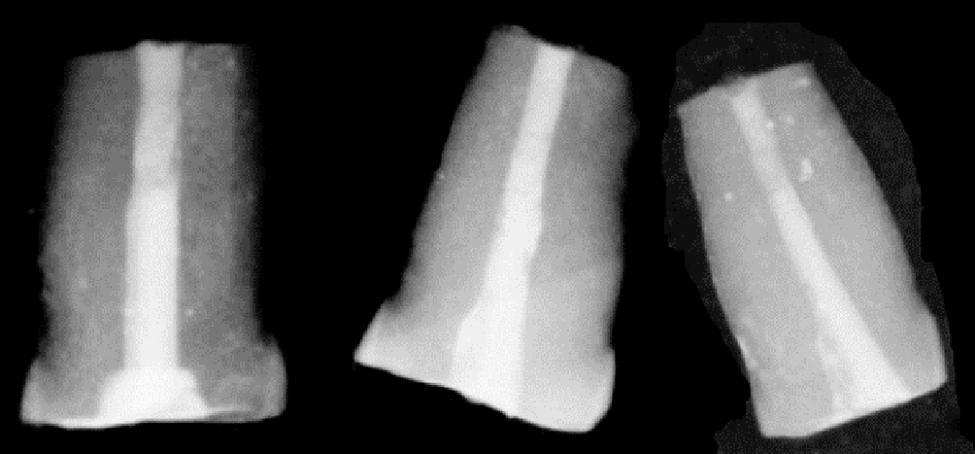
Radiographic view of the roots following the application of MTA and CC mixed with blood in the root-end cavity

Human blood was obtained from the blood bank. The blood was kept in a sterile blood tube, internally coated with spray-dried tripotassium ethylenediaminetetraacetic acid (K2EDTA; Vacuette, Greiner BioOne, Kremsmünster, Austria) to prevent clotting. The MTA and CC powders were mixed with blood in 2:1 ratio (2 units of powder mixed with 1 unit of blood), and the consistency of the mixture was the same in both groups, and for all teeth and they were placed into cavities using a #60 hand plugger (Shenzhen Denco medical Ltd., China) by the same operator. and conformation radiographies were taken immediately after that (Fig. 2).

**Fig. 3:**
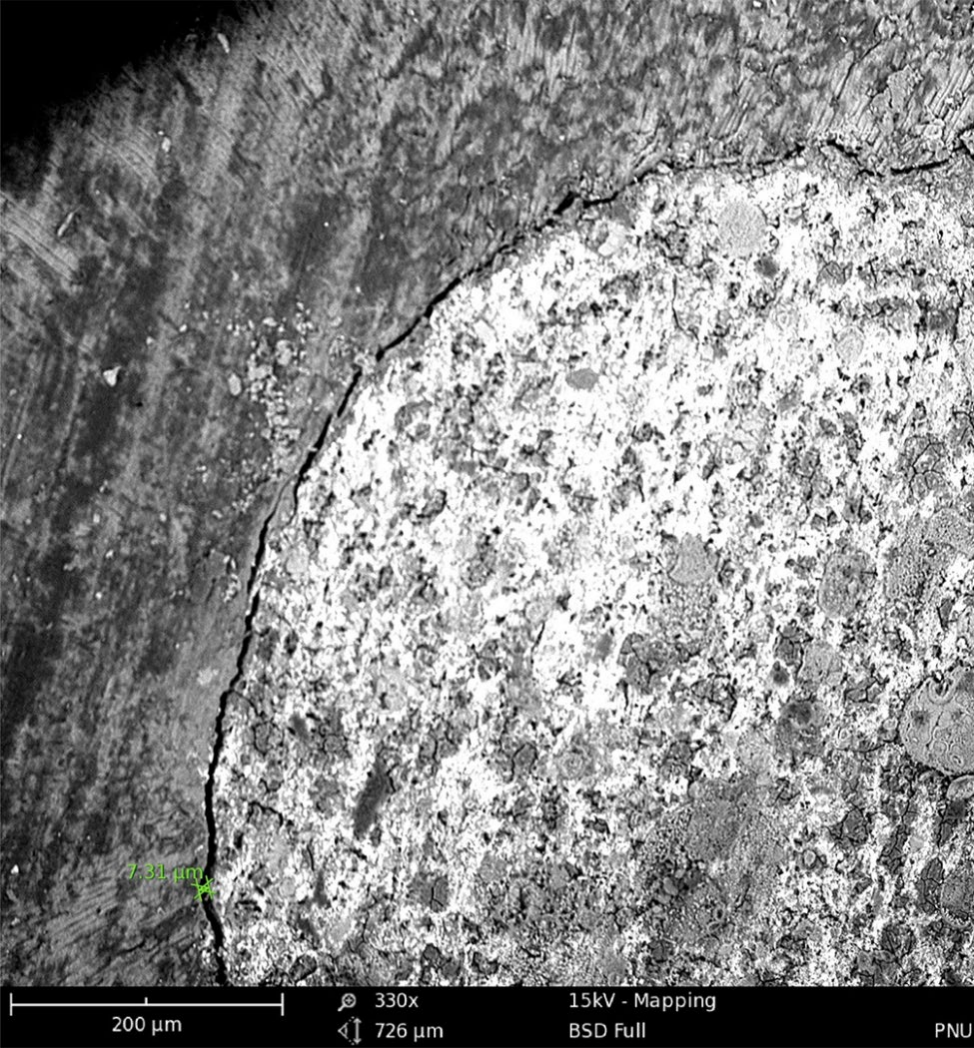
SEM micrograph of the interface of CC and dentinal cavity wall at the apex (green mark indicates the gap size between the CC and canal wall in micrometers)

**Fig. 4:**
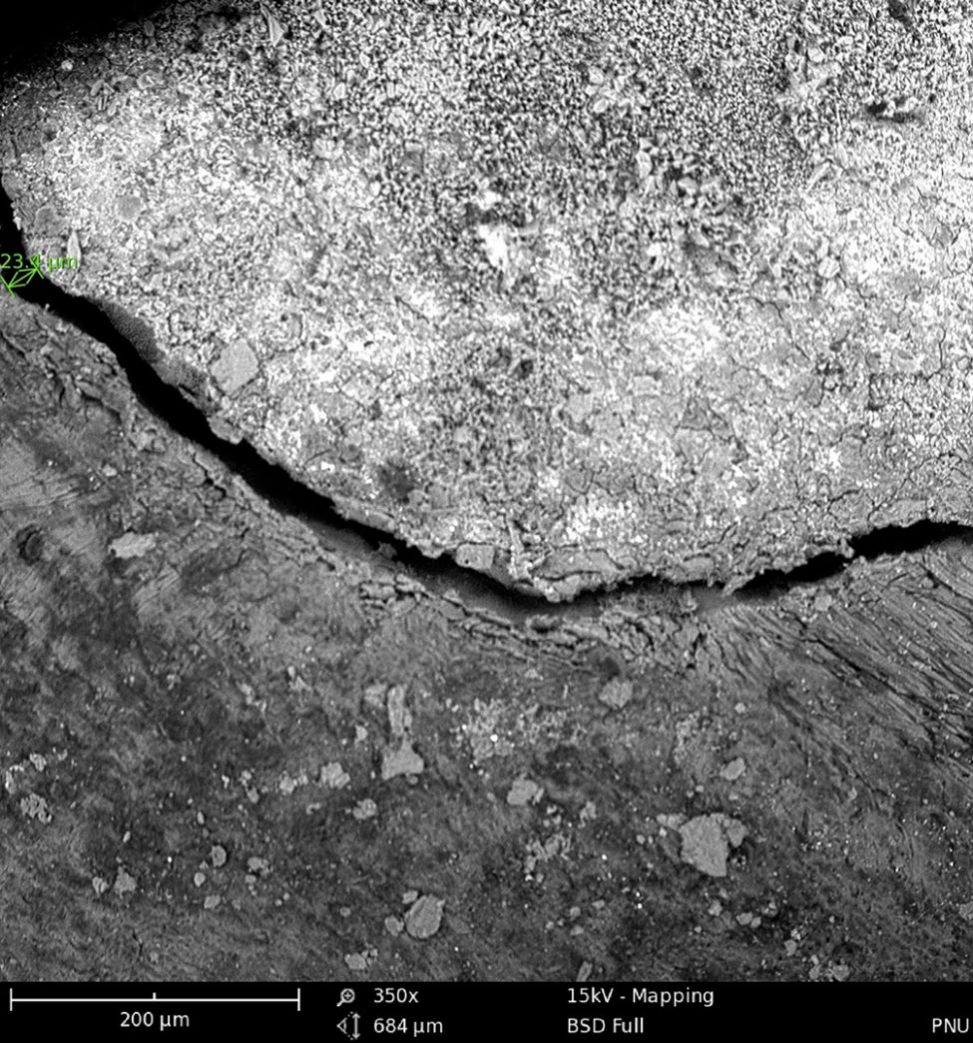
SEM micrograph of the interface of MTA and dentinal cavity wall at the apex (green mark indicates the gap size between the MTA and canal wall in micrometers)

The teeth were then incubated at 37 °C for 24 h. Next, in each group, the roots were transversely sectioned at 1.5 mm distance from the apex with a diamond disc under copious water irrigation. Afterwards, the largest gap between the root-end filling material and dentinal cavity wall in the transverse section was measured at the apex of the remaining root using SEM($\approx$×350) (Phenom G2 pro x, Phenom World BV, the Netherlands) (Figs. 3 and 4). The gap size was reported in micrometers (µm). All experiments and scorings have been performed by one and the same operator.

### Statistical analysis

**Diagram 1:**
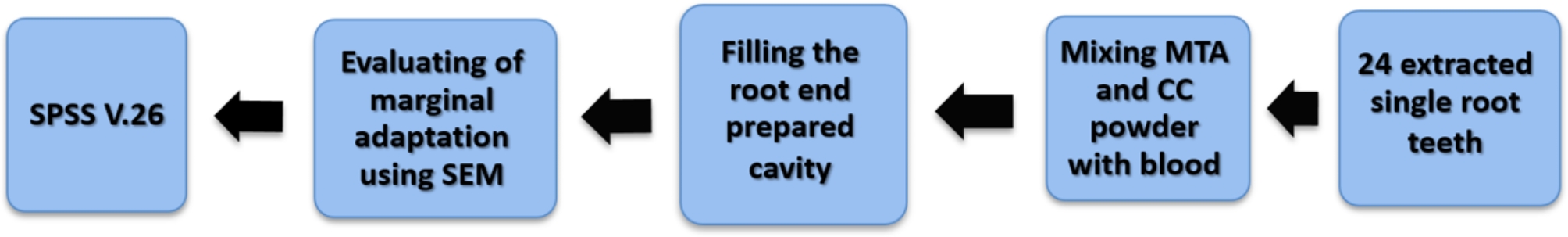
An overview of the experimental procedure

Data were analyzed by SPSS version 26 (SPSS Inc., IL, USA). The normality of data distribution was assessed by the Kolmogorov-Smirnov test, which confirmed normal distribution of data (P > 0.05). Thus, the two groups were compared regarding gap size by t-test. Level of statistical significance was set at 0.05. (diagram 1)

## Results

A gap existed between the root-end filling material and canal wall in all specimens in both groups. Table 1 presents the measures of central dispersion for the gap size between the root-end filling material and cavity wall in the MTA and CC groups. The lowest observed gap score (4.72 μm) was noted in one specimen in the CC group, and the highest observed gap score (23.40 μm) was found in one specimen in the MTA Angelus group.

**Table 1:** Measures of central dispersion for the gap size (µm) between the root-end filling material and cavity wall in the MTA and CC groups (n = 12)

Group	Mean	Median	Std. deviation	P value*
Cold ceramic	8.98	9.07	2.29	0.000
MTA	16.26	17.20	4.10	0.000

*t-test, P < 0.001

According to t-test, the mean gap size was significantly smaller in the CC group than the MTA Angelus group, indicating significantly superior marginal adaptation of CC (P < 0.001).

## Discussion

This study assessed the effect of blood contamination (by mixing the powder with blood, instead of the respective liquid) on marginal adaptation of CC and MTA Angelus using SEM. The results showed that the mean gap size was significantly smaller in the CC group than the MTA Angelus group, indicating significantly superior marginal adaptation of CC group. Thus, the null hypothesis of the study was rejected.

Hasheminia et al. [24] compared the sealing ability of MTA and CC in dry, saliva-contaminated, and blood-contaminated environments using the dye penetration technique with 2% methylene blue. They reported that in blood-contaminated environment, the magnitude of dye penetration in the CC group was significantly smaller than that in the MTA group, indicating significantly superior sealing ability of CC. However, in dry and saliva-contaminated environments, the difference between the CC and MTA was not significant. They concluded that CC has a significantly superior performance regarding apical seal compared with MTA in blood-contaminated environments, which was in agreement with the present findings, although the present study had a different methodology. Mokhtari et al. [22] assessed the marginal adaptation of MTA and CC following mixing the powder and liquid according to the ratios recommended by the manufacturers. They measured the largest gap at the root-end filling material and root canal wall interface after 24 h using SEM, and reported significantly smaller marginal gap in the CC group than the MTA group; however, the difference between the two groups did not reach statistical significance. Torabinejad et al. [25] compared the marginal adaptation of MTA, Amalgam, Super-EBA, and IRM as root-end filling materials using SEM and reported that MTA yielded significantly higher marginal adaptation compared with the other three materials. Xavier et al. [26] assessed the seal ability of Super-EBA, MTA, and Vitremer by the dye penetration technique (with silver nitrate) and SEM. The SEM results showed significantly smaller gaps in the MTA group. Also, the dye penetration test revealed the highest microleakage in the Vitremer group, and showed that the performance of MTA and Super-EBA was significantly superior to that of Vitremer. Jovanović and Bajkin [27] compared the marginal adaptation of MTA, Biodentine, and amalgam as root-end filling materials. They measured the gap size between the root-end filling material and root canal wall in longitudinal sections using SEM, and found no significant difference among the three materials, although the gap size was the smallest in MTA and the largest in amalgam. El-Sherief et al. [28] evaluated the marginal adaptation of glass ionomer, MTA Flow and a new formulation known as MTA Harvard as root-end filling materials using SEM. After 24 h, the gap size was significantly smaller in MTA Flow, indicating significantly superior marginal adaptation of MTA Flow compared with other tested materials. Bansal et al. [29] compared the marginal adaptation of MTA, Biodentine, and MTA Plus as root-end filling materials by measuring the gap size using SEM. The gap size was significantly smaller in Biodentine, indicating its significantly superior seal ability and marginal adaptation, compared with the other two materials.

In all the above-mentioned studies, the mean gap size between the MTA and root canal wall ranged from 1.05 to 8.17 μm. In all the aforementioned studies, the powder and liquid were mixed according to the manufacturer’s instructions. However, in the present study, the MTA powder was completely mixed with blood, instead of water, and the mean gap size in this group was measured to be 16.26 μm, which is almost double the largest gap size for MTA reported in the literature. This finding highlights the negative effect of blood contamination on sealing ability of MTA, which should be taken into account in clinical practice.

In the abovementioned studies, the mean gap size between the CC and root canal wall under SEM ranged from 2.72 to 6.09 μm, and in all of them, the CC powder and liquid were mixed according to the manufacturer’s instructions. However, in the present study, the CC powder was completely mixed with blood, and the mean gap size was calculated to be 8.98 μm. Although this finding also indicates the negative effect of blood contamination on sealing ability of CC, but the impact was not as great as that on MTA, and the obtained mean gap size for CC was close to the highest gap size reported for normally mixed MTA in the literature.

Mokhtari et al., [30] in their in vitro study compared the seal ability of CC and MTA by using a bacterial leakage model (Enterococcus faecalis) over a 30-day period. They noticed microbial leakage in 20% of CC, and 35% of MTA specimens and found no significant difference in this regard between the two groups. Karobari et al. [31] compared the seal ability of Neo MTA Plus, ProRoot MTA, and Biodentine using the dye penetration method (1% methylene blue). They found a significant difference among the three groups and reported that glass ionomer had the poorest performance while Biodentine and Neo MTA Plus equally had the best performance. Nepal et al. [32] compared the apical microleakage of glass ionomer, MTA, and Biodentine by the dye penetration test using 2% methylene blue, and showed that after 72 h, MTA and Biodentine had significantly lower leakage than glass ionomer, but the difference between MTA and Biodentine was not significant. In another study, Kararia et al. [33] evaluated the seal ability of MTA and Retroplast by the dye penetration test using 1% Rhodamine B. They reported that after 1 week, leakage was noted in 95% of all specimens in both groups, with no significant difference between them. Paulo et al. assess the impact of blood contamination on the push-out bond strength achieved with three distinct biomaterials applied into root canal. The findings of the study reveal that TotalFill exhibits the most prominent values of push-out bond strength, subsequently followed by Biodentine, and ultimately by MTA [17].

Khedmat et al. [21] assessed the biocompatibility of MTA and CC in an in vitro study using periodontal ligament fibroblasts. They showed that both materials were highly biocompatible, and fibroblasts favorably attached to the surface of both materials. Modaresi et al. [12] confirmed optimal biocompatibility of both MTA and CC. In another study, Modaresi [13] showed optimal seal ability and short primary setting time of CC and discussed that CC is a suitable alternative to MTA as a root-end filling material. The present results indicated that in case of presence of blood contamination, CC may be a more suitable option than MTA.

This study had an in vitro design, which limits the generalizability of the results to the clinical setting. Clinical studies are required to obtain more reliable results. Also, the present study assessed the marginal adaptation after 24 h. Future studies are required to assess the marginal adaptation over longer periods of time. Furthermore, other properties of CC such as its push-out bond strength ,compressive strength and solubility should be assessed after blood contamination.

## Conclusion

within the limitation of this in vitro study, it can be concluded that following complete blood contamination of powder, CC showed significantly superior marginal adaptation than MTA Angelus as shown by SEM assessment.

## Data Availability

The data used to support the findings of this study were supplied by corresponding author under license and data will be available on request. Requests for access to these data should be made to corresponding author.

## Declarations

All methods were carried out in accordance with relevant guidelines and regulations.

## List of abbreviations

***CC***: Cold Ceramic
***MTA***: Mineral Trioxide Aggregate
***SEM***: Scanning Electron Microscopy
